# A Systematic Approach to Preclinical Trials in Metastatic Breast Cancer

**DOI:** 10.4172/2167-7700.1000204

**Published:** 2016-05-30

**Authors:** OM Rashid, D Maurente, K Takabe

**Affiliations:** 1 Holy Cross Hospital Michael and Dianne Bienes Comprehensive Cancer Center, 4725 North Federal Highway, Fort Lauderdale, FL 33308,USA; 2 Massachusetts General Hospital, 55 Fruit St, Boston, MA 02114, USA; 3 University of Miami Miller School of Medicine, 1600 NW 10th Ave, Miami, FL 33136, USA; 4 Florida Atlantic University Charles E. Schmidt College of Medicine, 777 Glades Road, Boca Raton, FL 33431, USA; 5 Virginia Commonwealth University School of Medicine and the Massey Cancer Center, Division of Surgical Oncology, Department of Surgery, Richmond, VA, USA; 6 Roswell Park Cancer Institute, Elm and Carlton Streets, Buffalo, NY 14263, USA

## Abstract

The process of developing new agents for therapy against breast cancer is inefficient and relies on animal models to screen for efficacy for preclinical studies. However, there has been limited validation of these models, despite the increasing costs in the rapidly growing era of personalized medicine and targeted therapy. Recently, there have been multiple studies which have critically evaluated animal models for breast cancer drug discovery. We recently reviewed the transgenic, xenograft, and syngeneic murine breast cancer models, the ectopic, orthotopic and intravenous methods of cell implantation, tumor gene expression profiles, as well as the ethics of animal experimentation, and we provide important information for investigators in this challenging field. Because of the complexities of treating breast cancer and the increasing costs of developing new agents, the choice of the appropriate murine model must carefully consider each model available, including the tumor gene expression profile. Such a critical approach to the *in vivo* portion of drug development will further increase the efficiency of breast cancer drug research and development.

## Introduction

Breast cancer represents a leading cause of cancer death for women in the U.S. and accordingly, billions of U.S. dollars have been invested over decades in search of a cure [1,2]. Our review highlights the resources expended in preclinical trials which rely heavily on murine models of breast cancer to screen for efficacy [3]. Although the field of breast cancer has made significant advances, especially in the era of targeted therapy and personalized medicine, there has not been a limited evaluation of these models in this context until recently [3]. This review includes an evaluation of transgenic, xenograft, and syngeneic murine breast cancer models, the ectopic, orthotopic and intravenous methods of cell implantation, tumor gene expression profiles, as well as the ethics of animal experimentation [3]. In order to more efficiently and effectively undertake preclinical breast cancer trials, investigators must strategically consider to what extent the model available is the appropriate system for testing their hypothesis in question. In fact, even when experimentation is performed in a humane fashion, in compliance with all regulatory standards of animal experimentation, investigators are ethically obliged to carefully consider what experiments they perform on animals so that results are meaningful and animals are not wasted [3]. Therefore, aside from the scientific imperative to understand the limitations of the model system used to test scientific hypotheses, there is an ethical imperative as well [3]. This review highlights the strengths and weakness of each model to further guide investigators.

**Figure 1:**
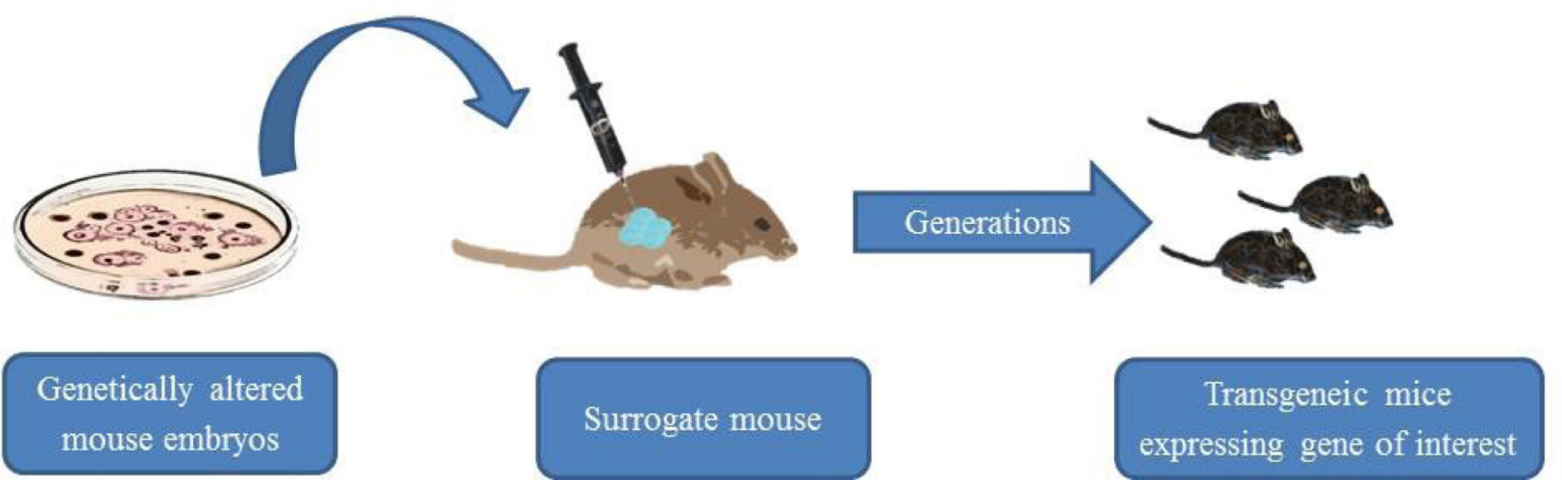
Transgeneic murine model for human breast cancer research.

Transgenic models offer the benefit of producing tumors spontaneously, which is a system that mimics human carcinogenesis. Furthermore, it allows for testing hypotheses related to specific genetic targets with implications for cancer treatment, especially in the era of targeted therapy. While such a system seems attractive to investigators, it does have several limitations which warrant consideration. First, the tumors produced in transgenic models rarely metastasize [3,4]. Second, the tumors produced in transgenic models often lose their estrogen receptor status and are not morphometrically stable long-term [3,4]. Third, the transgenic system is limited to only the genes tested, which is a simplification of the human condition and it places limitation on translatability [3,4]. Fourth, transgenic tumors take months to form spontaneously, which increases the resources needed to conduct studies [3,4]. While transgenic models do play a role in breast cancer research, it is important to weigh these considerations when designing studies and interpreting results (Figure 1).

Xenograft models offer an attractive system for breast cancer research because of the opportunity to test hypotheses using human tissue implanted into immune deficient mice: the benefit of human tissue without the risks to human subjects. Orthotopic xenograft mouse models replicate the course of human tumor progression, allow for the investigation of targeted molecular therapeutic interventions, and require only a single biopsy for multiple tests [5]. However, there are important limitations to consider which can confound the result. First, implantation of human breast cancer tissue into mice eliminates the cancer-host interactions which occur in the human disease [3]. Instead, the human cancer tissue interacts with the microenvironment and cells of the mouse host. Therefore, it is unclear whether the results obtained from such a model would be the same if the human tumor were interacting with the native human host. Second, xenograft models utilize immune deficient mice in order to avoid immune mediated rejection of the human tumor tissue [3].

**Figure 2:**
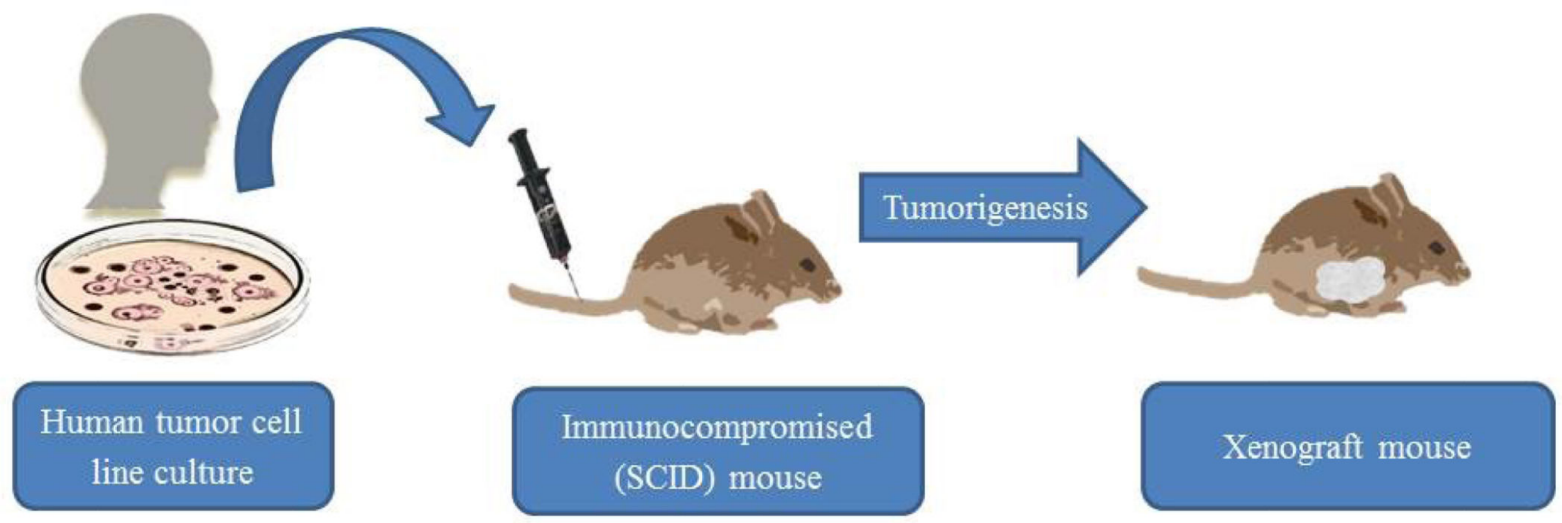
Xenograft murine model for human breast cancer research.

This variable is important to consider when interpreting results and designing experiments because of the increasingly important role of the host immune system in the management of cancer, which xenograft models ignore. Third, the xenograft models utilize a sample of a human tumor, not the entire tumor. Because of tumor heterogeneity, it is difficult to be certain which clones within the tumor are being tested in the model or how that may reliably translate in humans [3]. Fourth, tumors produced by xenograft models do not undergo a predictable pattern of progression, which can limit reproducibility and translatability of results [3]. Although the prospect of utilizing human tumor tissue in mice to test novel agents is appealing at first glance, it is important for investigators to understand these limitations and pursue parallel lines of investigation to test their hypotheses (Figure 2).

**Figure 3:**
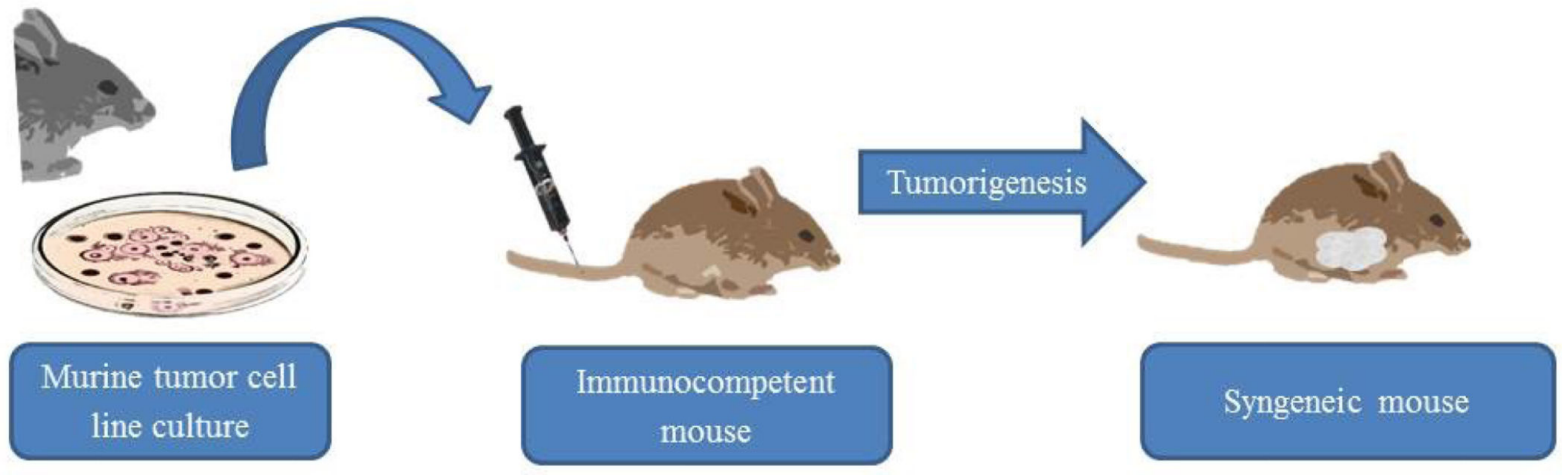
Syngeneic murine model for human breast cancer research.

Considering the limitations of the above mentioned models, implantation of mouse derived mammary gland adenocarcinoma into immune intact syngeneic mice provides a system that addresses some of the previously discussed limitations. First, using a single cell line eliminates the problems of tumor heterogeneity and quality control of tumor samples [6–9]. However, the obvious limitation is that the cell line is a murine cancer, not a human cancer, and therefore translatability is limited. Second, utilizing mouse derived mammary gland adenocarcinoma in the same genetic background mice allows for implantation into immune intact mice [6–9]. The benefit of this system is that hypotheses related to the immune system can be evaluated [6–9]. In addition, tumor-host interactions can also be evaluated [6–9]. The limitation, however, remains that there are important differences between mouse immunity and human immunity, as well as between tumor-host interactions in this artificial system compared to human disease [6–9]. If an investigator decides that implantation of syngeneic mouse derived mammary gland adenocarcinoma is the appropriate model (Figure 3), the next question to address is the appropriate implantation method.

Before deciding on which implantation method to utilize, investigators must first decide what their hypothesis is testing. Is a primary tumor required or only metastatic lesions or both? Do translatable clinical endpoints such as cancer progression and survival matter? Because the use of syngeneic mouse derive mammary gland adenocarcinoma makes use of host-tumor interactions, many have advocated orthotopic implantation into the mammary gland [6–9].However, there has been controversy regarding orthotropic implantation versus ectopic implantation, as well as whether orthotropic implantation into the mammary gland should be percutaneously or directly into the small gland under direct vision, and whether to implant into the chest or abdominal mammary glands [6–9]. Although it has been argued that ectopic implantation into the subcutaneous flank is faster and makes no difference in outcome, recent publications have demonstrated significant differences in tumor progression, clinical endpoints, and tumor gene signature between these methods, even when using the same cell lines in the same genetic background mice [6–9]. Compared with chest implantation under direct vision injection, subcutaneous tumors grow more slowly with a fibrous capsule do not progress along the Halstead pathway to regional axillary lymph nodes before distant metastasis, and have statistically significant differences in gene signature, without any significant delays in time to performing or learning to perform the procedure [6–9]. In addition, the majority of the differences in gene signatures included known candidates of basic science and clinical research [6–9]. Furthermore, it has been shown that direct vision injection more reliably produces implantation into the mammary gland with less variability between tumors and larger size than the percutaneous technique [6–9]. Finally, abdominal mammary gland implantation produces inguinal lymphatic spread and carcinomatosis by direct invasion of the abdominal cavity, compared to chest mammary glands which mimic human progression as summarized above [6–9]. While each method of producing primary tumors has its advantages and limitations (Table 1), the next question investigators must address is whether the presence of distant metastasis is important for testing their hypothesis.

When considering metastatic breast cancer models, investigators must consider what types of metastasis they wish to treat and whether the presence of the primary tumor is needed. With the presence of a primary tumor, investigators have the opportunity evaluate questions related to cross-talk between the primary tumor and metastatic lesions [6–9]. In fact, it has been shown that the gene signatures of the lung metastases that arise from the orthotopically implanted primary tumor are significantly different than the primary tumor from which it arose [6–9]. An alternative method of testing hypotheses on breast cancer metastasis is tail vein injection, where the mouse lungs are colonized with cancer cells and produce lung metastases [6–9]. The benefit of this method is that it quickly produces lung metastases without the presence of a primary tumor. Advocates of this method prefer it for cell lines in which it is difficult to form primary tumors, or in which the primary tumor does not metastasize in mice. Although histologically tail vein injection produces diffuse lung lesions as opposed to the solitary lung metastatic tumors that progress from orthotopic implantion, it has been shown that there is no significant difference in the genetic signatures of these lung lesions [6–9]. However, there is a difference in the ability to monitor clinical endpoints of progression. Even when using bioluminescence to quantify the tumor burden and monitor response to therapy, mortality in the tail vein injection model is often due to thromboembolic phenomena, rather than a gradual progression due to cancer progression [6–9]. While there is no perfect model that faithfully mimics human disease in mice, these considerations are important for investigators to take into account as they proceed with research (Table 2).

Currently, there is no consensus murine model for breast cancer research and drug development. Accordingly, there has been an intensive effort to develop patient-derived xenograft models and humanized mice with hope to create more ideal models for investigation than are currently available. Because there is no ideal model available, investigators must become experts on the methods available. They must understand what each model has to offer and choose the appropriate model in a disciplined fashion that takes all the nuances into account, including the gene signatures of the tumors they produce [7,8]. This review advocates such an approach and provides important information for all those interested in pursuing preclinical trials for breast cancer [3].

## Figures and Tables

**Table 1:** Comparison of murine models for human breast cancer research.

Summary of implantation sites used in murine mouse models			
Model	Description	Advantages	Limitations
Orthotopic	Exogenous introduction of genetic material to modify the expression of endogenous genes.	- Spontaneous tumor production - Solitary lung metastases	- Differences between murine tumor biology and human diseases.
Tail vein	Transplantation of human tumour cell line into immunocompromised mice.	- Rapid development of lung metastases	- Absence of primary tumor - Diffuse lung metastases
Subcutaneo us	Transplantation of murine tumour cells line into immunocomponent mice.	- Homogenous cell line - Intact tumor-host immune interaction - Quality control of samples	- Murine cell line - Murine tumor-host interaction

**Table 2:** Comparison of implantation methods in murine models for human breast cancer research.

Summary of implantation sites used in murine mouse models		
Model	Advantages	Limitations
Orthotopic injection under direct vision	- Replicates the course of tumor progression - Allows for analysis of primary tumor in native microenviroment - Develops solitary lung metastases - Develops larger size tumors with less variability	- Differences between murine tumor biology and human diseases.
Ectopic percutaneous injection		- Develops more slowly and within a fibrous capsule - Does not progress along Halstead pathway - Develops differences in gene signatures metastasis
Tail vein injection	- Rapid development of lung metastates - Does not differences in gene signature upon metastasis	- Absence of primary tumor - Develops diffuse lung metastates - Mortality 2° to thromboemboli - Difficult to monitor clinical endpoints
